# The dazed and confused identity of Agassiz’s land tortoise, Gopherus agassizii (Testudines, Testudinidae) with the description of a new species, and its consequences for conservation

**DOI:** 10.3897/zookeys.113.1353

**Published:** 2011-06-28

**Authors:** Robert W. Murphy, Kristin H. Berry, Taylor Edwards, Alan E. Leviton, Amy Lathrop, J. Daren Riedle

**Affiliations:** 1 Centre for Biodiversity and Conservation Biology, Royal Ontario Museum, 100 Queen’s Park, Toronto, ON Canada M5S 2C6; 2 State Key Laboratory of Genetic Resources and Evolution, Kunming Institute of Zoology, the Chinese Academy of Sciences, Kunming 650223; 3 U.S. Geological Survey, Western Ecological Research Center, 21803 Cactus Avenue, Suite F, Riverside, CA 92518 USA; 4 Arizona Research Laboratories, University of Arizona Genetics Core, 1657 E. Helen Street, 6 Room 111, Tucson, AZ 85721 USA; 5 Department of Herpetology, California Academy of Sciences, 55 Music Concourse Drive, San Francisco, CA 94118 USA; 6 Department of Agriculture and Environmental Science, Lincoln University, 904 Chestnut St., Jefferson City, MO 65101 USA

**Keywords:** Lectotype, *Xerobates*, *Gopherus lepidocephalus*, desert tortoise, recovery units, California, Arizona, Mexico

## Abstract

We investigate a cornucopia of problems associated with the identity of the desert tortoise, *Gopherus agassizii* (Cooper). The date of publication is found to be 1861, rather than 1863. Only one of the three original cotypes exists, and it is designated as the lectotype of the species. Another cotype is found to have been destroyed in the 1906 San Francisco earthquake and subsequent fire. The third is lost. The lectotype is genetically confirmed to be from California, and not Arizona, USA as sometimes reported. Maternally, the holotype of *Gopherus lepidocephalus* (Ottley & Velázques Solis. 1989) from the Cape Region of Baja California Sur, Mexico is also from the Mojavian population of the desert tortoise, and not from Tiburon Island, Sonora, Mexico as previously proposed. A suite of characters serve to diagnose tortoises west and north of the Colorado River, the Mojavian population, from those east and south of the river in Arizona, USA, and Sonora and Sinaloa, Mexico, the Sonoran population. Species recognition is warranted and because *Gopherus lepidocephalus* is from the Mojavian population, no names are available for the Sonoran species. Thus, a new species, *Gopherus morafkai* **sp. n.**, is named and this action reduces the distribution of *Gopherus agassizii* to only 30% of its former range. This reduction has important implications for the conservation and protection of *Gopherus agassizii*, which may deserve a higher level of protection.

Been dazed and confused for so long, it’s not true

Jake Grier Holmes, Jr. 1967 (not Jimmy Page 1968)

## Introduction

Often, systematics and taxonomy are clear cut. Species are described and they persist in recognition, either as being valid taxa or buried in a synonymy. That said, taxonomic chaos also occurs, often with respect to generic allocation, the validity of subspecies (Frost and Hillis 1990), and the recognition of species themselves. The taxonomy of the desert tortoise, or Agassiz landtortoise, is engulfed in errors. Some errors have now persisted for almost 150 years, and others are more recent in origin.

Berry et al. (2002) summarize data suggesting that the desert tortoise, *Gopherus agassizii* (Cooper), of the southern United States and northwestern mainland Mexico is a composite of at least two and possibly four species. They note that much work remains to be accomplished before formally recognizing any new species. This task is more complex than originally imagined, in part because of a convoluted taxonomy plagued with uncertainties and problems. Our reviews of several conundrums obtain the background data required to untangle a knot of confusion and make some decisions and recommendations. The greatest problem concerns the identities of true *Gopherus agassizii* and the enigmatic *Gopherus lepidocephalus* (Ottley et Velázques Solis).

### Date of publication of Cooper’s name Xerobates agassizii

**Figure 1.**
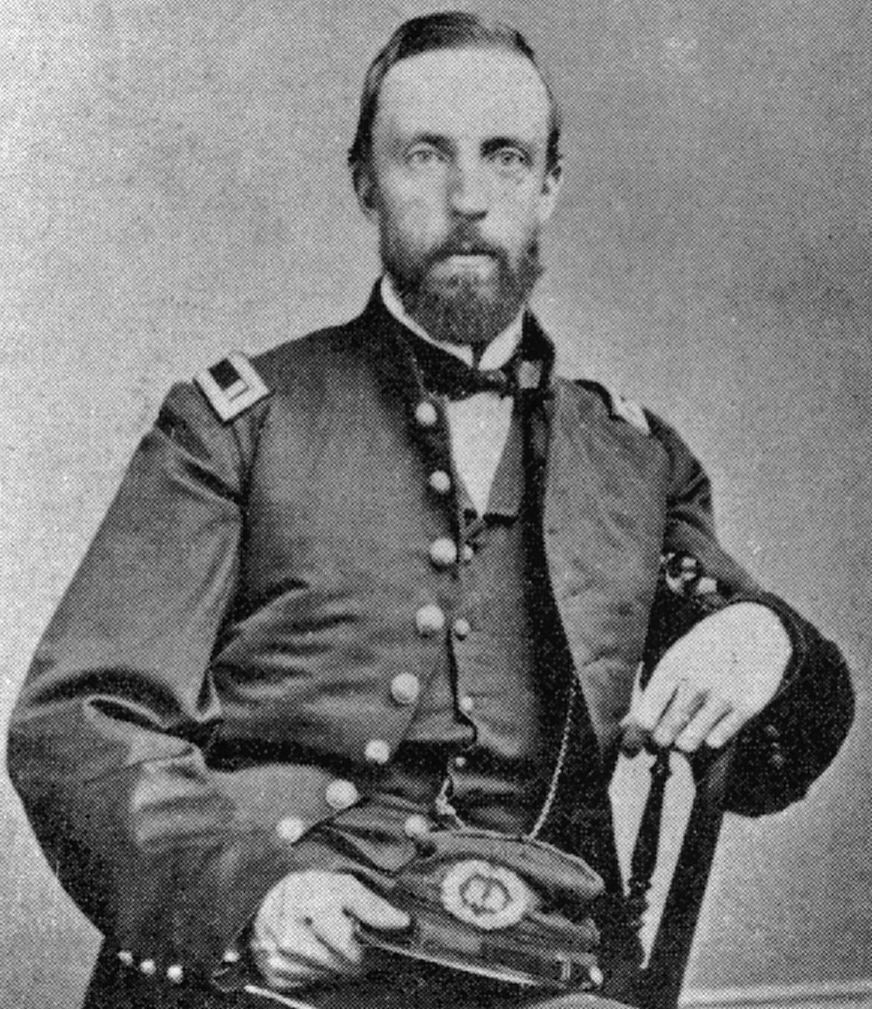
Portrait of Dr. James Graham Cooper, M.D. who discovered and described *Xerobates agassizii* (courtesy of the Archives of the California Academy of Sciences).

The discovery of *Gopherus agassizii* was first presented by James G. Cooper, MD (Fig. 1) as a new genus and species, *Xerobates agassizii*, the “Agassiz Land-Tortoise,” at the California Academy of Natural Sciences meeting of 7 July 1861 (Leviton and Aldrich 1997: 53). Shortly thereafter, in 1861, the description was published as a separate issue (termed a signature) of the Proceedings of the Academy. However, there is confusion about the date of publication. The collected Proceedings, series 1, volume 2 spanned the years 1858–1862 but was closed in 1863 when a dated title page, table of contents, and index were issued. The closing date has long been used in error for the description of *Xerobates agassizii* (e.g. Van Denburgh 1897; Cochran 1961; Auffenberg and Franz 1978; Crumly 1994; Reynolds et al. 2007), and this error may have been started by True (1881), who states the description was “…issued in 1863.” Curiously, in the same issue of the Proceedings, Cooper (1861) described *Athene whitneyi* (=*Micrathene whitneyi*, the elf owl) and *Helminthophaga luciae* (=*Oreothlypis luciae*,Lucy’s warbler), and historically these descriptions have been correctly credited to 1861, as have a series of botanical papers credited to Albert Kellogg (Leviton et al. 2010: 235–236). Although originally named as the Agassiz landtortoise, and not Agassiz’s land tortoise as given by Cooper (1870: 67) and quoted in error by True (1881), it was also once also called Agassiz’s Gopher (Yarrow 1883). Today the species is commonly referred to as the desert tortoise, a transliteration of *Xerobates* (xeros, Gr. dry; bates, Gr., one that walks, treads, haunts) that dates back to Van Denburgh (1897). This common name is also applied to other tortoises in the genus *Testudo*. Unlike Latinized names, common names do not enjoy precedence.

Documentation of the publication date has required a venture into the history of the Proceedings of the California Academy of Natural Sciences (see Leviton and Aldrich 1997, 2010). The Proceedings, started in September 1854, were initiallyissued in four or eight-page signatures, later expanding to 16-page signatures. Printing required about one to four months and the signatures were distributed by the Academy usually within four days of receipt from the printer. Volume 1 included signatures published between 4 September 1854 and 31 January 1858. Volume 2 contains contributions from 22 February 1858 to 15 December 1862, and it was closed in 1863. This volume is comprised of 15 numbered, 12- or 16-page signatures, the first eight of which are not dated but they were printed shortly after the last included dated meetings. For example, signature number 1 (pp. 1–16), which included all materials presented at meetings held between 22 February 1858 and 26 September 1859, but was otherwise undated, was printed between 26 September 1859 and, at the latest, 26 January 1860, but in all likelihood within days of 26 September 1859. This was followed by signature 2 (pp. 17–32) which reported on activities, including the text of papers presented, for the period 26 September to 24 October 1859, and so forth to signature 8 (pp. 110–124), which covered the meetings held between 15 April to 21 July 1861, the last, undated signature to be included in volume 2. After that, signatures were print-dated. Signature 9 (pp. 125–140), for the period 21 July to 19 August 1861, was dated December 1861; signature 10 (pp. 141–156), for the period 19 August through 1 December 1861, was dated April 1862, and so forth. Thus, signature 8, which contained the pages bearing Cooper’s original description of *Xerobates agassizii*, was printed and available for distribution no earlier than four days following the last meeting reported on in the signature, i.e., 21 July 1861, but no later and most likely weeks earlier than the print date of signature 9, which is given as December 1861. We propose that the official date of publication should be 25 July 1861.

### Type locality of Xerobates agassizii

Credited to multiple places, the type locality of *Gopherus agassizii* has been thoroughly confused. *Xerobates agassizii* was described on the basis of three “young” cotypes collected from the “mountains of California, near Fort Mojave” (Cooper 1861). In late 1860, Cooper, a medical officer in the Army, was assigned to report for duty at Fort Mojave, an Army fort located on the east bank of the Colorado River in northern Arizona (see Coan 1981: 100–106 for more details). In early December 1860, Cooper travelled to the Fort via a Quartermaster’s wagon train departing from Los Angeles. The group traveled via Cajon Pass and then across the Mojave Desert, reaching the Fort on 20 December. His time at the Fort was to be truncated by the onset of military action in the East—the Civil War—which led to the abandonment of the Fort on 28 May 1861. However, before leaving California for Fort Mojave, Cooper had already contacted Josiah Dwight Whitney, director of the California State Geological Survey. During his return trip from Fort Mojave to the Pacific Coast, beginning on 29 May 1861, Cooper prepared a report for Whitney describing the conditions of some of the areas through which he passed, including Pah-Ute Spring, Rock Spring, and Soda Lake (Soda Playa), which during his trip from Los Angeles to Fort Mojave he described as “in December the only warm part of the route east of Cajon Pass...” (Coan 1981: 104).

When Cooper finally reached Los Angeles, sometime during the second half of June 1861, he found a letter waiting for him from Spencer Fullerton Baird, then Assistant Secretary of the Smithsonian Institution, informing him that Whitney had expressed an interest in hiring him, Cooper, as the State Survey’s zoologist. Cooper arrived in San Francisco on 4 July 1861, met with Whitney, and then sent a letter to Baird stating that he had encountered two new birds at Fort Mojave as well as a new species of tortoise (Coan 1981: 105). In the letter, Cooper also informed Baird that he planned to describe the new tortoise in the Academy’s Proceedings, and perhaps jokingly asked, “…who shall I name it for, Agassiz?” (Cooper to Baird, 14 July 1861) (Coan 1981: 105).

By the time Cooper described the new tortoise, he had already been hired by Whitney as the California State Geological Survey’s zoologist. Cooper was also a member of the California Academy of Sciences, where he held the title Curator of Zoology in 1862. Curiously, the date of Cooper’s Academy membership has been as enigmatic as the information associated with some of the specimens he collected. According to the Academy’s membership list, Cooper became a member on 18 February 1867, at least six years after the dates with which we are concerned. But not only did Cooper attend the Academy’s meeting in the latter half of 1861, at one of which (7 July 1861) he presented his paper describing new species of Californian animals, in early 1862 he was elected Curator of Zoology, which could only have happened if he were already a member (Leviton and Aldrich 1997: 54).

Irrespective of Academy membership, Cooper’s collection near Fort Mojave was made before he was employed by the Survey. Once employed by the Survey, all specimens collected thereafter were treated as Survey property. Regardless, sometime after 1861, Cooper sent one of the cotypes, a juvenile, to Baird at the United States National Museum, Smithsonian Institution (USNM 7888). Although the specimen supposedly was collected by Cooper in March 1861, the collecting locality has been credited to multiple places. Cochran (1961), and Auffenberg and Franz (1978) gave the locality of this cotype as “Utah Basin, Mojave River.” However, according to Cochran (1961) the catalog gives the locality as “Solado Valley, California.” Reynolds et al. (2007: 32) provide some clarification. The USNM catalogue states: “The original parchment label attached to USNM 7888 lists the locality as Soda Valley, but the original catalog record has Solado Valley.” Further, an old label in the jar with the specimen and Yarrow (1883) give the locality as Solado Valley. Regarding Utah Basin, Reynolds et al. (2007) further state “This information is not in the catalog record, and we have been unable to determine why she (Cochran) included it in the locality for this specimen.” Certainly, the type locality occurs within the “Mountains of California, near Fort Mojave” (Cooper 1861), and most likely in Soda Valley (today also known as Soda Playa). The USNM catalogue states that the specimen was collected in March 1861 (Reynolds et al. 2007). Cooper passed through the area on at least two occasions, first in early December 1860 and again in early June 1861 enroute to and on his return from Fort Mojave, Arizona, about 83 km. Unfortunately, none of Cooper’s writings for that period have survived.

### Fate of the two other cotypes

The fate of the remaining two specimens Cooper had collected is also confused. Reynolds et al. (2007: 32) state “Two other syntypes were originally in the collection of the California State Geological Survey and later deposited in the California Academy of Sciences”, and that “CAS 7141 and CAS 7142 … were the likely syntypes …” However, these two tortoises were collected on 11 March 1905 by John Carlson, and thus could not have been the two missing cotypes of *Xerobates agassizii* Cooper, 1861. Given that most of the records of the California Academy of Sciences were destroyed in the San Francisco earthquake and subsequent fire of 1906, no written record of a transfer of the cotypes to the Academy exists, although the catalogue of the herpetological specimens, started by Van Denburgh in 1894, was saved and exists today (see below).

One of the three possible cotypes was likely deposited in the herpetological collections of the Academy. The herpetological collections that accumulated between 1853 and 1894 were not cataloged until 1894, when John Van Denburgh came to the Academy and initiated the formal catalog of amphibians and reptiles. Possible cotype CAS 3567, catalogued as “*Gopherus agassizii*,” was collected by Cooper. The specimen was likely catalogued in 1896, well after Stejneger (1893) made the generic change from *Xerobates* to *Gopherus*. The specimen was undated and the locality was originally recorded as being “Arizona.” However, a note by Van Denburgh in the Department’s catalog states that Cooper said the tortoise came from the Mojave Desert, California. This specimen may or may not have been one of the three cotypes. Regardless, tortoise CAS 3567 was destroyed in the earthquake and subsequent fire in 1906 as were three other specimens, CAS 3568, 3269, and 3570, all shown as *Gopherus agassizii*. Of the latter three, CAS 3568, listed as a shell, was collected at “Crater Summit, Mojave Desert,” by Oscar Brown, but without a date. Numbers CAS 3569, a shell, and CAS 3570, a skeleton, have “original numbers”, but it is not known whether these numbers were either field numbers or numbers from an earlier cataloging effort, the records of which no longer exist; no other data exist. If CAS 3567 was one of the cotypes, then the whereabouts of the third juvenile specimen remains a mystery. In this scenario, it is possible that the third cotype was retained by the California State Geological Survey and was subsequently lost or destroyed. There is a remote possibility that a syntype was deposited elsewhere. For instance, some of the Survey’s paleontological collections formed the nucleus of the Museum of Paleontology’s collections at the University of California, Berkeley (UCMP) (Lipps 2004: 220), but part of the collection went to Harvard (MCZ) when Survey Director Whitney returned to the university, and a portion went to the Academy of Natural Sciences in Philadelphia (ANSP) with William Gabb. However, none of these collections have specimens of *Gopherus agassizii* collected in the 1860s or otherwise transferred by Cooper. Of course, it is also possible that the specimen was shipped elsewhere, but that seems unlikely.

### Description of Xerobates lepidocephalus and taxonomic views on its validity.

Ottley and Velázques Solis (1989) described a new species, *Xerobates lepidocephalus*, from the Cape Region of Baja California Sur, Mexico. Ecologically, the species occurs on sloped or hillside areas and it is not reported to live in burrows. This habitat choice closely resembles that of tortoises living in the Sonoran Desert, specifically those tortoises occurring east and south of the Colorado River. These tortoises, called Sonoran desert tortoises (Van Devender 2002), differ substantially from tortoises in the Mojave Desert. In general, Sonoran tortoises live in rock crevices on steep slopes and hill tops (Riedle et al. 2008) and Mojave desert tortoises live in burrows in valleys and on alluvial fans (Berry et al. 2002). Morphologically, *Gopherus lepidocephalus* is most similar to tortoises on Tiburon Island off the coast of Sonora, Mexico and the species was considered to be a junior synonym of *Gopherus agassizii* by Crumly and Grismer (1994).

### Generic instability

The generic allocation of the Agassiz land-tortoise, the desert tortoise, has occasionally changed. Cope (1875) transferred *Xerobates agassizii* to the genus *Testudo*, as *Testudo agassizii*, in his checklist of North American amphibians and reptiles but without comment or justification. Presumably, this determination followed the generic allocation of Gray (1870) and certainly this was not an oversight as Cope (1880) repeated the generic allocation for *Gopherus berlandieri*. The next taxonomic change was made by Stejneger (1893) in his discussion of the fauna of Death Valley. He considered the Californian tortoise to be distinct from *Gopherus berlandieri* and to belong to the North American genus *Gopherus* Rafinesque 1832, as “*Gopherus agassizii* (Cooper)” (Stejneger 1893: 160). This generic allocation was stable for almost 100 years.

Nomenclatural stability for *Gopherus agassizii* was maintained until Bramble (1982) revised the genus using both extant and extinct species. He discovered two groups and erected the genus *Scaptochelys* for *Gopherus agassizii* and *Gopherus berlandieri*. The type species was designated as *Xerobates agassizii* Cooper, 1863 [sic]. Thus, *Gopherus agassizii* was referred to as *Scaptochelys agassizii*. Shortly thereafter, Bour and Dubois (1984) reported that *Scaptochelys* was a junior synonym of genus *Xerobates* Agassiz, 1857, whose type species was *Xerobates berlandieri* Agassiz, 1857, by subsequent designation (Brown 1908). Because Bramble (1982) resolved *Scaptochelys agassizii* as the sister group of *Xerobates berlandieri*, Bour and Dubois (1984) referred *Scaptochelys agassizii* back to *Xerobates agassizii* Cooper, 1863 [sic].

In terms of generic allocation, Crumly (1994) aptly notes that the genus *Xerobates* cannot be diagnosed morphologically owing to intraspecific variation. Thus, he refers *Xerobates agassizii* back to *Gopherus agassizii* (Cooper, 1861). Symplesiomorphies are used by Bramble (1982) to define *Scaptochelys*, a practice that contravenes the principles of phylogenetic systematics. Although morphological evidence does not unite *Gopherus agassizii* and *Gopherus berlandieri*, molecular evidence does (Lamb and Lydeard 1994). And although it is possible to recognize *Xerobates* for the extant species *Gopherus agassizii* and *Gopherus berlandieri*, the phylogenetic relationships among extinct species (Reynoso and Montellano-Ballesteros 2004) preclude monophyly of the two genera. Thus, *Xerobates* should not be recognized.

### Species instability

Mertens and Wermuth (1955) and Wermuth and Mertens (1961) were unimpressed by the extent of morphological differentiation among North American *Gopherus* and impressed by the reports of hybrids. While recognizing long-term isolation, they recognized only one species, *Gopherus polyphemus*, stating “Da sich die einzelnen Formen der Gopherschildkröten äußberlich nur wenig unterscheiden, deutlich geographisch vikariieren und mehreren Veröffentlichungen zufolge auch zu verbastardieren scheinen, sind sie hier als Unterarten aufgeführt” (Wermuth and Mertens 1961: 172). In doing so, they considered *Gopherus agassizii* to be a subspecies of *Gopherus polyphemus*, *Gopherus polyphemus agassizii* (Cooper). Their taxonomic arrangement was rarely, if ever, followed.

### More than one species

**Figure 2.**
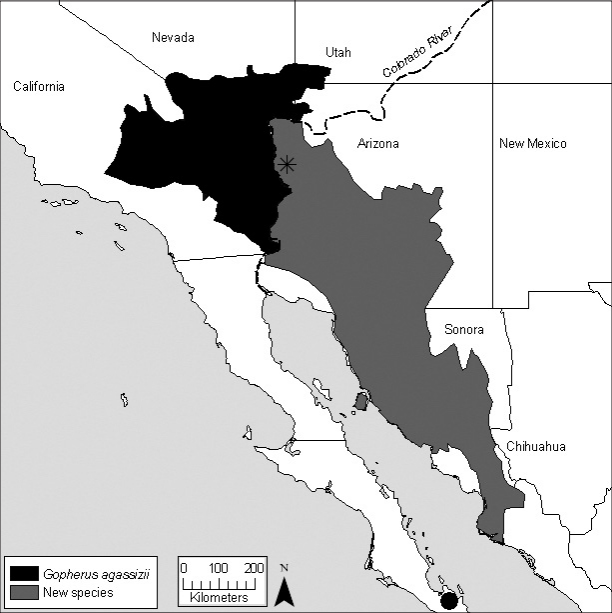
Distribution of the desert tortoises aligned with *Gopherus agassizii*. The locality of BYU 39706 from Baja California Sur is shown as a black dot. The location of the hybrid population described in McLuckie et al. (1999) is shown as a star.

Berry et al. (2002) summarize evidence for the existence of at least two species of desert tortoises, and support is still mounting (Table 1). Evidence includes either fixed or statistically significantly differences in microsatellite DNA alleles (Murphy et al. 2007; Engstrom et al. 2007; Edwards et al. 2011), differences in maternal lineages as evidenced by mitochondrial DNA (Lamb et al. 1989; Lamb and Lydeard 1994; Edwards 2007), significant behavioral and ecological differences (Berry et al. 2002), and perhaps significant differences in longevity and growth strategies (Curtin et al. 2009). The exception is one small, geographically restricted zone where the two forms of tortoises hybridize (Fig. 2; McLuckie et al. 1999; Edwards et al. unpublished data). The two forms are thought to have been isolated from 5 to 6 Ma (Lamb and Lydeard 1994; Lamb and McLuckie 2002) As currently conceived (Fritz and Havaš 2007), *Gopherus agassizii* is best viewed as a composite of at least two and possibly as many as four species (Berry et al. 2002).

**Table 1.** Summary of morphological, physiological, and ecological characteristics that differ between populations of desert tortoises from the Mojave and Sonoran deserts.

Character	Mojave Desert	Sonoran Desert	Reference
Morphology-shell shape			
Width of shell at mid-bridge	significantly wider shell		Germano 1993
Length of gular scutes	Significantly longer gular scutes		Germano1993
		Significantly shorter length of projection of anal scutes	Germano 1993
General shape of shell	California: box-like, high-domed; Utah: box-like, low-domed, shorter plastron	Flatter, pear-shaped	Weinstein and Berry 1989
Geographical distribution	North and west of the Colorado River	South and east of the Colorado River	
Habitats occupied Topography	Predominantly valleys and alluvial fans	Predominantly slopes and rocky hillsides	U.S. Dept. of the Interior, Fish and Wildlife Service 1994; Van Devender 2002
Vegetation types	Mojave Desert: Saltbush scrub, creosote bush scrub, desert scrub, tree yucca woodland	Sonoran Desert: Arizona upland, thornscrub, desert grassland	U.S. Dept. of the Interior, Fish and Wildlife Service 1994, 2010b; Van Devender 2002
Egg production			
Mid-line carapace length (mm MCL) at first reproduction	176 (Germano), 178 (Turner et al. 1987)	220	Turner et al. 1984, 1986, 1987; Germano 1994b; Henen 1994; Averill-Murray 2002, Averill-Murray et al. 2002, Curtin et al. 2009
Oviposition time (range)	April to mid-July	Early June to early August	Turner et al. 1986; Averill-Murray et al. 2002
Number of clutches/yr	0–3	0–1	Turner et al. 1986; Averill-Murray et al. 2002
Number of eggs per year	5–16	1–12, avg. ~5	Turner et al. 1986, 1987; Henen 1994; Karl 1998; Mueller et al. 1998; Wallis et al. 1999
Proportion of females ovipositing/yr	0.67–1.0; typically 1.0	0.36–0.80; typically < 1, based on one study (Averill-Murray)	Turner et al. 1986; Henen 1997; Mueller et al. 1998; Wallis et al. 1999; Averill-Murray 2002

Two species of desert tortoise can be recognized after a nomenclatural conundrum is solved. The population in the Mojave Desert that occurs north and west of the Colorado River, and the population in the Colorado Desert of California (Fig. 2; Berry et al. 2002), will bear the name *Gopherus agassizii* (Cooper, 1861) unless unequivocal data proves otherwise. Tortoises that occur east and south of the Colorado River will require at least one name. The true identity of *Gopherus lepidocephalus* remains a problem. It is possible, albeit seemingly unlikely, that this species is native and endemic to the Cape Region of Baja California, yet it is also possible that the tortoise represents a translocation from mainland Mexico, and perhaps from northern Sonora, Mexico (Crumly and Grismer 1994), or elsewhere. If the population in Baja California Sur is native to northern Sonora, Mexico, then the name *Gopherus lepidocephalus* (Ottley & Velázques Solis, 1989) will apply to tortoises south and east of the Colorado River currently known as *Gopherus agassizii* and irrespective of the type locality being non-native. Further, it is possible that another species is associated with tropical deciduous forests in southern Sonora and northern Sinaloa, Mexico (Lamb et al. 1989), and, if true, then it is also possible that the name *Gopherus lepidocephalus* applies to this potential species. Finally, and of some concern, it is possible that the species is a translocated hybrid population because hybrid individuals are exceptionally common in the ex situ, captive population (Edwards et al. 2010).

To evaluate the validity of *Gopherus lepidocephalus* and to confirm the geographic origin of *Gopherus agassizii*, we obtained mitochondrial DNA sequences from both type specimens. This kind of analysis could not detect hybrids because the mitochondrial genome is inherited only maternally. However, if *Gopherus lepidocephalus* has its origin in the Mojave Desert, then the name will persist as a junior synonym of *Gopherus agassizii* regardless of whether it is a hybrid or not. Alternatively, if the maternal lineage is from a Sonoran desert tortoise, then the possible hybrid state would create another problem to be solved. Finally, if the lineage was new and divergent, then perhaps *Gopherus lepidocephalus* was native to the peninsula.

## Materials and methods

Tissue samples (leg muscle) were dissected from the lectotype of *Gopherus agassizii* (Cooper, 1861) (USNM 7888) and the holotype of *Gopherus lepidocephalus* (Ottley & Velázques Solis, 1989) (Brigham Young University [BYU] 39706). Genomic DNA was extracted from approximately 10 mg of tissue. The lectotype of *Gopherus agassizii* was likely preserved in ethanol yet the holotype of *Gopherus lepidocephalus* was initially well-preserved in formalin. Subsequently, both specimens were stored in 70% ethanol. To remove fixatives, tissues were washed twice in PBS, pH 7.2 (50 mM potassium phosphate, 150 mM NaCl) as recommended in the DNA Easy Extraction Kit (Qaigen) for tissue exposed to formalin. Subsequently, higher yields of DNA were achieved using our standard extraction method, rather than the DNA Easy Extraction Kit, as follows: digestion of the tissue was carried out at 52 °C in a lysis buffer (Tris 6.06g, Na2EDTA 0.93g, NaCl 5.85g and SDS 1.0g, 500ml ddH2O, pH 8.5) and spiked daily with 12.5 µl of proteinase K (Roche) until the tissue sample was completely digested (5–7 days). Purification used two washes with phenol:chloroform:isoamyl alcohol followed by a final wash of chloroform:isoamyl alcohol.

### Primer design

Using the alignment of Murphy et al. (2007), primers were designed for a 423 bp fragment that was diagnostic for haplotypes of *Gopherus agassizii*. The forward primer GoCytL (5’-CAATTCGATTCTTCCTAGTAGC-3’) was located in the NADH3 gene and reverse primer GoCytH (5’- GGCTGAGAAGGATAGTATTAGTATTGG-3’) located on ND4. Attempts to amplify the holotype sample of *Gopherus lepidocephalus* (BYU 39706) failed after numerous attempts using these two primers. Because DNA exposed to formalin is prone to degradation and fragmentation (Bucklin and Allen 2004), several internal primers were designed and used in various combinations until amplification was successful. Eventually we amplified a 225 bp fragment using the original GoCytL forward primer and a new internal reverse primer (LepidoNd3h3: 5’-TTGGTGTCATTTTGATAGCCGTGAAG-3’) that straddles the tRNAArg and ND4L genes; one bp was not confidently resolved.

### PCR amplification

Each PCR was carried out in 25ul volume on a DNA Engine PTC-200 (MJ Research). Due to a low concentration of the template (2–5ng/µL), 30µl of the DNA extraction was concentrated via standard ethanol precipitation. Subsequently, the reagents for the PCR were used to resuspend the pellet. The reagents included 0.8µl of 10mM dNTP, 1µL of each 10µM primer, 2.5 µl 1x PCR buffer (1.5 mM MgCl2; Fisherbrand), and 0.75 U Taq DNA polymerase (Fisherbrand). Cycling parameters were 94 °C for 2 min, 39 cycles of 94 °C for 30s, 55 °C for 45s, 72 °C for 45s and a final extension at 72 °C for 7 min.

To verify amplicons, a 25 µl of the PCR product was run out in a 1.5% agarose gel stained with ethidium bromide and visualized under ultraviolet light. Bands were excised and purified by spinning in a filter tip (Sorenson; 75–30550T) that was set in a 1.7µl Eppendorf tube. Samples were centrifuged at 16.1G for 10 min. We used 4µl of the cleaned PCR product for sequencing on an ABI3100 (Applied Biosystems) using a ¼ reaction of Big Dye 3.1 recommended by ABI (Applied Biosystems).

### Negative controls

DNA was extracted a minimum of three times for the lectotype of *Gopherus agassizii* and once for the holotype of *Gopherus lepidocephalus*. To avoid any possibility of cross contamination, final extractions were done in isolation of one another. Amplification and sequencing were also done independently for both strands. Desert tortoise sequences were confirmed using a BLAST search of the NCBI database.

### Sequence analysis

The sequence data were aligned by eye using CLUSTALW (Thompson et al. 1994) against fragments used by Murphy et al. (2007), which were downloaded from GenBank (accession No. DQ649394–DQ649409), because (1) the target region mostly contained encoding sequences, and (2) the length of the fragment precluded the necessity of computer-assisted alignments. Nucleotide divergences against the most similar sequence were merely counted. The fragment was too short and the levels of divergence too small to be used for meaningful tree constructions.

## Results

**Figure 3.**
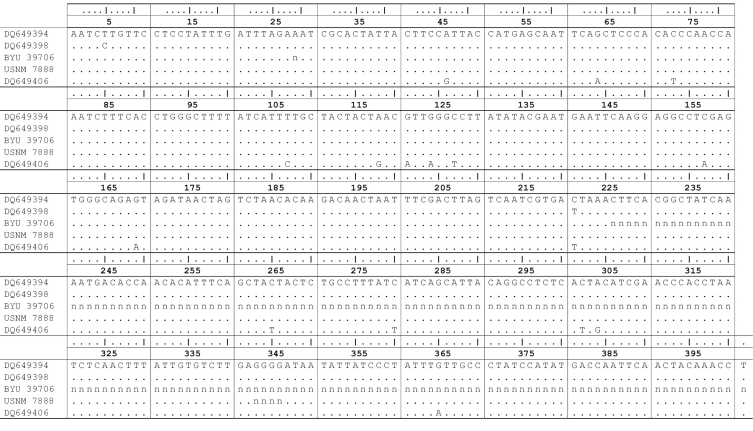
Implied alignment of the mitochondrial DNA sequence data spanning the partial genes NADH3, tRNAArg and ND4L from tortoises of the *Gopherus agassizii* complex. BYU 39706 is the holotype of *Gopherus lepidocephalus*. USNM 7888 is the lectotype of *Gopherus agassizii*. GenBank sequence DQ649394 is the sequence of *Gopherus agassizii* in widespread group A of Murphy et al. (2007), DQ649398 is from narrowly distributed group B, and DQ649406 is a specimen of *Gopherus morafkai* from Tucson, Arizona. “n” indicates unresolved or ambiguous base pairs.

We resolved a 224 bp fragment for *Gopherus lepidocephalus* and 401 aligned nucleotides from the lectotype of *Gopherus agassizii* including 4bp that were not confidently resolved. Attempts to sequence a larger fragment from *Gopherus lepidocephalus* failed. The shorter sequence was located completely within the larger. The aligned sequences were identical (Fig. 3). A BLAST search in GenBank revealed that the sequences were identical to the most common haplogroup in tortoises from the Mojave Desert—group A in Murphy et al. 2007 (e.g. GenBank Acc. No. DQ649394; Fig. 3). Group A was detected throughout the Mojave Desert except in the Northeastern Mojave Recovery Unit. The sequences ofthe lectotype of *Gopherus agassizii* (USNM 7888) and holotype of *Gopherus lepidocephalus* differed from group B (e.g. GenBank Acc. No. DQ649398; Fig. 3) by only 2 of 224 bp (0.9%); the longer sequence of the lectotype differed at the same 2 nucleotide positions only (0.5%) from group B (Fig. 3). In contrast, the sequence ofthe lectotype of *Gopherus agassizii* (USNM 7888) differed from Sonoran desert tortoises (e.g. GenBank Acc. No. DQ649406; Fig. 3) by 16 of 397 bp (4.0%). The shorter fragment from both the lectotype of *Gopherus agassizii* and the holotype of *Gopherus lepidocephalus* (BYU 39706) differed from Sonoran desert tortoises by 11 of 224 bp (4.9%). Thus, at least the maternal lineages of both type specimens were from the Mojave Desert, and not the Sonoran Desert. This discovery did not exclude the possibility of *Gopherus lepidocephalus* being a hybrid individual.

Several observations suggested the absence of DNA contamination. First, amplification of DNA from the two type specimens resulted in differing fragment lengths. Primers used for the lectotype of *Gopherus agassizii* did not amplify DNA from the holotype of *Gopherus lepidocephalus*. Thus, it is exceptionally unlikely that contamination occurred between these two species. Neither type specimen had DNA extracted along with other samples of *Gopherus*; all comparative samples were downloaded from GenBank. Thus, cross-contamination outside of this project was not possible. Finally, DNA extracted in isolation of the other type precluded the possibility of contamination. Consequently, all evidence suggested that the sequence data were obtained from the respective specimens.

Given that only one of the cotypes is known, we propose the following designation for *Gopherus agassizii*:

### Agassiz’s Desert Tortoise Gopherus agassizii

(Cooper, 1861)

http://species-id.net/wiki/Gopherus_agassizii

**Figure 4.**
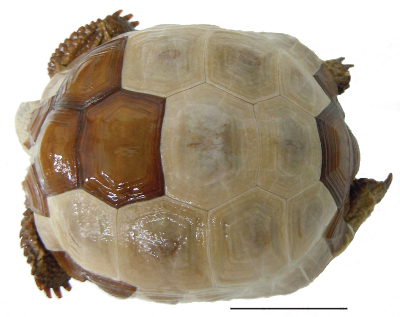
Dorsal view of the lectotype of *Gopherus agassizii*, USNM 7888. Black bar is 3 cm.

Figs 4 [Fig F5] [Fig F6] [Fig F7] 8

#### Lectotype.

 USNM (National Museum of Natural History, Smithsonian Institution) 7888; terra typica restricta: California, San Bernardino County; Mountains of California, near Fort Mojave; Soda Valley (very approximately 35°6'N, 116°6'W). We restrict the type locality to that published by Cooper (1861) and the parchment tag associated with the specimen. References to other localities, including Solado Valley, an apparent synonym of Soda Valley, should be considered to be in error.

The evolutionary species concept (Simpson 1961; Wiley 1978) suggests that the Sonoran population of the desert tortoise should be recognized as a new taxon. Frost and Hillis (1990) effectively argue that subspecies should not be recognized for continuously distributed species; we agree. Given these two observations, at least two species of desert tortoise should be recognized. The DNA sequence data exclude application of the available name *Gopherus lepidocephalus* for the Sonoran Desert population of *Gopherus* that occurs west and south of the Colorado River and they confirm that the lectotype of *Gopherus agassizii* is from the Mojave Desert, and not Arizona. Because no names are available for the tortoise population occurring in the Sonoran Desert south and east of the Colorado River, we describe it as a new species.

**Figure 5. F5:**
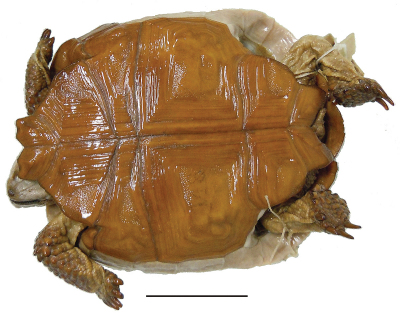
Ventral view of the holotype of *Gopherus agassizii*, USNM 7888. Black bar is 3 cm.

**Figure 6. F6:**
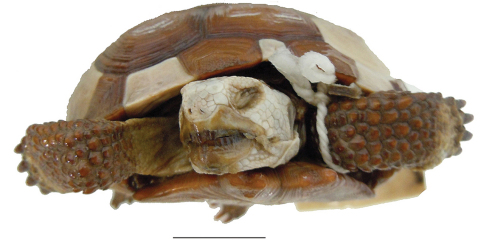
Anterior view of the holotype of *Gopherus agassizii*, USNM 7888. Black bar is 3 cm.

**Figure 7. F7:**
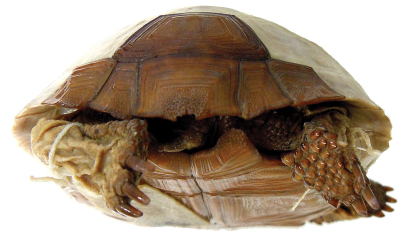
Posterior view of the holotype of *Gopherus agassizii*, USNM 7888.

**Figure 8.**
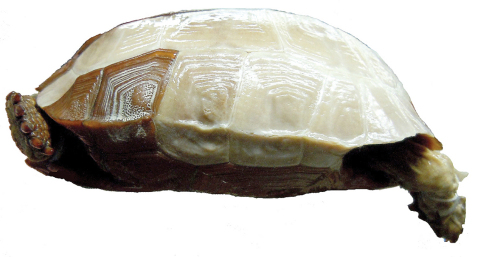
Left, lateral view of the holotype of *Gopherus agassizii*, USNM 7888.

### Morafka’s Desert Tortoise Gopherus morafkai sp. n.

urn:lsid:zoobank.org:act:B4A14033-BD75-4D90-BFD0-CA3C76FB17EF

http://species-id.net/wiki/Gopherus_morafkai

**Figure 9.**
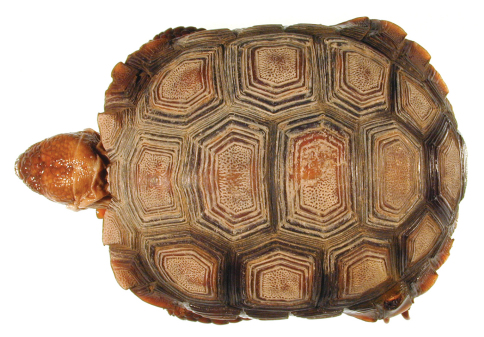
Dorsal view of the holotype of *Gopherus morafkai*, CAS 33867.

Figs 9 [Fig F10] [Fig F11] [Fig F12] [Fig F13] [Fig F14] 15

Xerobates agassizii Cooper 1861 (partim)
Testudo agassizii (Cooper 1861) (partim). Generic reassignment by Cope (1875)
Gopherus agassizii (Cooper 1861) (partim). Generic reassignment by Stejneger (1893)
Scaptochelys agassizii (Cooper 1861) (partim). Generic reassignment by Bramble (1982)
Xerobates lepidocephalus Ottley et Velázques Solis 1989. In error by Crumly and Grismer (1994)

#### Holotype.

 CAS (California Academy of Sciences) 33867; juvenile from Tucson (approximate location 32°7'N, 110°56'W, elevation 948 m), Pima County, Arizona, U.S.A, collected on 9 July 1912 by H. Brown and preserved in ethanol.

#### Paratypes.

 ROM (Royal Ontario Museum) 47501, formerly CAS 13165, an immature tortoise collected by H. Brown from 20 miles (32 km) west of Tucson, (presumably the Roskruge Mountains, Pima County) Arizona, USA (approximate location 32°7'N, 111°18'W, where tortoises occur today), on 9 March 1908, received at CAS alive on 23 March 1908, and died 8 July 1908; CAS 34263, a juvenile collected by J.R. Slevin in the Catalina Mountains (Santa Catalina Mountains), foothills at west end of mountains, Pima County, Arizona, USA on 15 May 1912 (approximate location 32°21’N, 110°57’W). Specimens are preserved in ethanol.

#### Diagnosis.

 All of the species of *Gopherus* and their hybrids can be easily diagnosed using molecular data. Morphologically, *Gopherus morafkai* can be separated from both *Gopherus flavomarginatus* and *Gopherus polyphemus* in having relatively smaller front feet. Whereas the distance from the bases of the first to fourth claws is the same on all feet in *Gopherus morafkai*, in the latter two species the distance from the bases of the first and third claws on the forelimb is about the same as the distance between the bases of the first and fourth claws on the hindlimb (Auffenberg and Franz 1978). The diagnosis of living specimens of *Gopherus morafkai*, *Gopherus berlandieri* and *Gopherus agassizii* can be impossible in captive tortoises because of extensive hybridization (Edwards et al. 2010) and because of abnormalities in shell, head and limb integument from poor nutrition (Donoghue 2006). However, in non-hybrid individuals, *Gopherus morafkai* can be separated from *Gopherus berlandieri* in having a rounded snout when viewed from above as opposed to a wedge-shaped snout in *Gopherus berlandieri* (Auffenberg and Franz 1978). Further, in *Gopherus morafkai* the gular projections do not normally diverge, and it has a single axillary scale preceding each bridge, yet in *Gopherus berlandieri* the gular projections often diverge and the axillary scales are often paired. Morphologically, *Gopherus morafkai* can be separated from *Gopherus agassizii* in having a relatively narrower shell, shorter gular scutes, shorter projections of the anal scutes and in having a flatter, pear-shaped carapace (Table 1). Ecologically, whereas *Gopherus agassizii* predominantly occurs in valleys and alluvial fan topography, *Gopherus morafkai* prefers slopes and rocky hillsides (Riedle et al. 2008), including animals of the isolated population in northwestern Arizona (McLuckie et al. 1999).

#### Description of holotype.

**Figure 10. F10:**
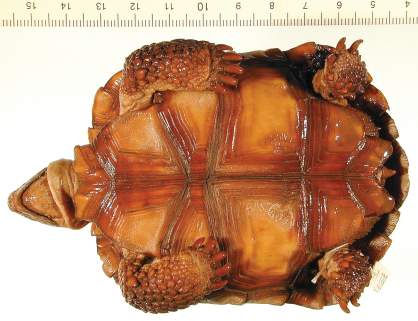
Ventral view of the holotype of *Gopherus morafkai*, CAS 33867.

**Figure 11. F11:**
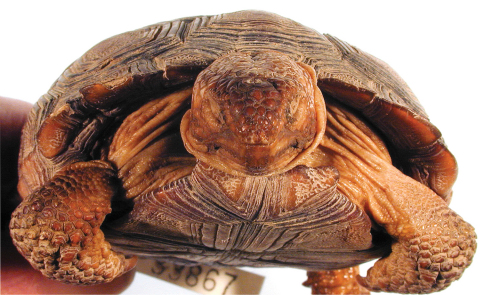
Anterior view of the holotype of *Gopherus morafkai*, CAS 33867.

**Figure 12. F12:**
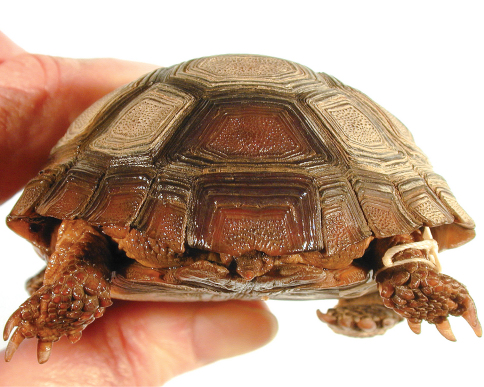
Posterior view of the holotype of *Gopherus morafkai*, CAS 33867.

**Figure 13. F13:**
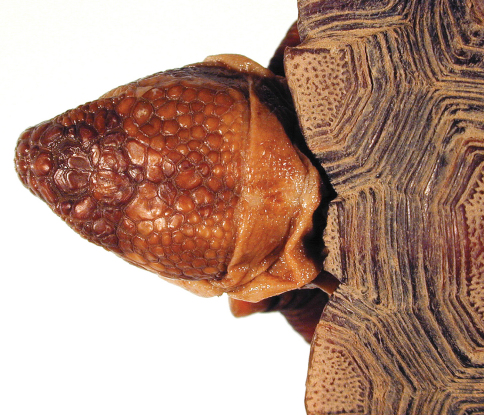
Detail of head scales of the holotype of *Gopherus morafkai*, CAS 33867.

 A juvenile, with straight-line carapace length at midline (MCL) = 86.5 mm, maximum carapace length is 88.5, curved carapace length from free edge of nuchal scute to that of supracaudal scute = 118 mm, maximum plastron plastron length from tip of gular horn to tip of anal scutes = 86 mm, midline plastron length from gular notch to anal notch = 78 mm, maximum height of shell at 3rd vertebral scute = 40 mm, width at 3rd marginal scute = 64 mm, maximum midbody width = 69, maximum width at 7th marginal scute = 73 mm, and head length from tip of snout to posterior edge of supraoccipital condyle = 25 mm (Figs 8, 12). Eleven marginal scutes present on both right and left edges of carapace. Supracaudal scute single, undivided. Five toenails present on each forelimb, four toenails on each hind limb (Fig. 9). Third nail of each hind limb longer than others. Two enlarged, raised scales present on anterior ventral surface of foreleg of which the ventral-most scale is larger, more protruding than others. Scales on head smooth, asymmetrical, larger anteriorly at snout, becoming much smaller in temporal area (Fig. 12). Areolae and 7 to 8 growth laminae present on all scutes. In alcohol, the color of areolae and adjacent two growth laminae on carapacial scutes (Figs 8, 11) predominantly dark reddish brown grading to reddish black on laminae at or near seams between scutes. Small areas of areolae on 2nd and 3rd vertebral scutes and left 1st costal scute yellowish brown or copper. Color of areolae on plastron light olive brown grading to dark yellowish brown on 2nd through 4th laminae. Laminae at and adjacent to the seams dark reddish brown with a few areas of dark red. Head and neck multi-colored (Figs 8, 9, 13): neck and throat very pale yellowish brown and very pale brown. Dorsal and lateral surfaces of head darken from parietal to frontal scales (Fig. 12). Skin in the axillary and inguinal areas also lighter in coloration, becoming reddish brown to dark reddish brown on lower limbs and pads of feet (Fig. 10). Nails golden brown at tips.

**Figure 14. F14:**
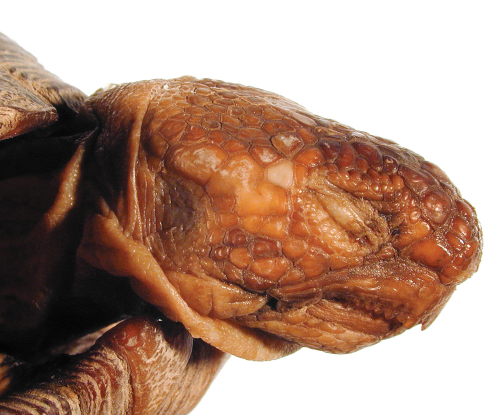
Right, lateral view of the head of the holotype of *Gopherus morafkai*, CAS 33867.

**Figure 15.**
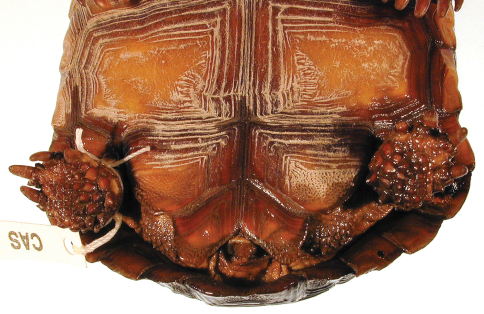
Detailed view of the anal scutes of the holotype of *Gopherus morafkai*, CAS 33867.

#### Coloration of the species in life.

 Coloration of *Gopherus morafkai* varies considerably by size and age as well as by location. Adult tortoises generally have hues and chromas of the integument in dark colors, e.g., very dark greyish brown, dark brown, very dark brown, olive brown, dark olive brown, reddish brown, dark reddish brown, dark grey, black, and occasionally to rarely xanthic tones (GretagMacbeth 2000). Neonates and young juveniles tend to be bi-colored, with orange to reddish areolae and reddish brown to dark brown laminae. As the juveniles age, they become darker. Coloration of limb scales tends to mirror that of the shell. Based on observations of the authors, the protected skin in axillary and inguinal areas is generally in lighter colors for all sizes and ages of tortoises.

#### Variation.

 Variation in coloration and morphology deserve further research with respect to location, vegetation and soil types, as well as by size, sex, and age of the tortoise. All future studies should include genetic documentation of non-hybrid specimens.

#### Distribution.

*Gopherus morafkai* occurs naturally east and south of the Colorado River in Arizona, as well as in Sonora, including Tiburon Island, and Sinaloa on the west side of the Sierra Madre Occidental, Mexico (Berry et al. 2002). The species appears to have been recently introduced from Sonora into at least one home in La Paz, Baja California Sur, Mexico as pets, where it successfully reproduced (Patricia Galina, personal communication to RWM). It likely occurs as introduced individuals or populations in North America and possibly elsewhere, although in this case many individuals are likely hybrids of *Gopherus morafkai* x *agassizii*.

#### Natural history.

*Gopherus morafkai* occurs in upland habitats in the Sonoran Desert scrub (Brown et al. 1979) with rocky outcrops and palo verde-saguaro cactus communities and ecotonal desert grasslands (Van Devender 2002). Within these habitats, *Gopherus morafkai* is generally found along rocky slopes, or bajadas, of desert mountain ranges, with breeding populations occurring as high as 1,420 m elevation and individual observation records occurring to 2,380 m (Flesch et al. 2010). The species typically occupies excavated or eroded burrows underneath rocks or boulders. Consequently, geology and resultant burrow availability among mountain ranges is an important determinant in regulating population density (Averill-Murray et al. 2002a, b).Low density populations of *Gopherus morafkai* also occur along alluvial fans and in intermountain valleys, where individuals utilize desert washes and associated caliche caves as shelter sites (Riedle et al. 2008; Grandmaison et al. 2010). These peripheral populations provide important genetic linkages between disjunct mountain ranges (Edwards 2003; Edwards et al. 2004; Averill-Murray and Averill-Murray 2005).

*Gopherus morafkai* exhibits both a spring (mid-March to May) and a late summer activity period (late July to late September). Activity patterns are rainfall-dependent, with increased activity related to increased precipitation during the late summer monsoons (Averill-Murray et al. 2002b). Monsoonal storms within the range of G.* morafkai* result from warm season winds pushing tropical moisture northwards from the Pacific Ocean and northern Mexico (Turner and Brown 1994). Female activity begins earlier than male activity in the spring, possibly because females might need to forage to develop shelled eggs before oviposition in June and July (Averill-Murray et al. 2002a). Activity is higher for both sexes during late summer monsoons, with courtship and breeding occurring in July–September (Averill-Murray et al. 2002a). Females develop ovarian follicles before entering brumation in the fall (Henen et al. 2000). The follicles probably mature in the spring with oviposition shortly afterwards (Henen et al. 2000; Averill-Murray 2002). Clutch size ranges from 1–12 eggs with a mean of 5.7 eggs (Averill-Murray 2002).

Female *Gopherus morafkai* mature at larger sizes (220 mm carapace length) (Averill-Murray 2002) than does *Gopherus agassizii* (176–190 mm carapace length) (Turner et al. 1986; Germano 1994a; Karl 1998). Clutch sizes between the two species are similar (Averill-Murray 2002), but *Gopherus morafkai* only produces 1 clutch every 1–2 yr (Averill-Murray 2002) while *Gopherus agassizii* may produce 1–3 clutches every year (Turner et al. 1986; Wallis et al. 1999). Harsher, more arid climates in the Mojave Desert may have led to increased female reproductive investment to offset hatchling and juvenile mortality (Heppell 1998; Hellgren et al. 2000), but information is limited for juvenile tortoises of both species.

Annual survivorship for juvenile *Gopherus morafkai* at three sites in Arizona ranged from 0.84 to 0.93 (Averill-Murray et al. 2002a). Adult survivorship was high (0.89–0.97). Seasonal differences in mortality reflected seasonal differences in activity patterns (Riedle et al. 2010). Adult survivorship was similar between both species (Table 1), although little was determined about hatchling or juvenile survivorship. Primary sources of mortality for *Gopherus morafkai* in Arizona included the following: 1) falls related to steep rocky habitat; 2) being overturned during combat and mating rituals; and 3) predation by mountain lions, *Puma concolor* (Riedle et al. 2010). Prehistorically, Native Americans ate Mojave and Sonoran tortoises (Schneider and Everson 1989) and historically, Native Americans and Mexicans hunted the tortoise for food (Cooper 1861; Cox 1881), although Cooper (in Cronise 1868: 480; see True 1881) reported that they were “not very well flavored.”

#### Etymology.

 The new species is a patronym for the late Professor David Joseph Morafka in recognition of his many contributions to the biology and conservation of the species of *Gopherus* and his unsurpassed way of facilitating research, even among researchers with very different perspectives.

## Discussion

### Few paratypes

We designated only two of many possible paratypes to exclude the possibility of hybrid individuals in the type series. Hybrid animals would confound the identity of *Gopherus morafkai* (Edwards et al. 2010). To this end, we specifically selected individuals collected from near the turn of the 19th Century from one of the oldest western North American herpetological collections, the California Academy of Sciences. The intent was to select paratypes collected before the development of major trans-desert highways that followed mass-produced automobiles, which in turn facilitated interspecific translocations. The future documentation of variation in the species, which should be accomplished within the context of geographic and habitat variation, must be restricted to wildcaught individuals genetically confirmed to be non-hybrids. Unfortunately, this may exclude the use of many formalinfixed animals in museum collections. Such investigations could delineate morphological characters useful in identifying F1 and other hybrid individuals.

### Common names

Common names do not enjoy precedence and they can create much unnecessary confusion. Historically, the species of *Gopherus* were commonly referred to simply as gophers, a word that normally refers to mammals. Now that *Gopherus morafkai* is recognized, the desert tortoise requires two common names. *Gopherus agassizii* could be referred to as the Mojavian desert tortoise, yet this is inaccurate because the species also occurs within the Sonoran Desert of California. Therefore, we prefer to call it Agassiz´s desert tortoise. This name also serves to retain the original designation of Cooper (1861). Similarly, *Gopherus morafkai* occurs in the Mojave Desert of Arizona, the Sonoran Desert of Arizona, USA and Sonora, Mexico and in Sinaloan thornscrub, but not in the Mojave and Sonoran deserts of California. Therefore, the term Sonoran desert tortoise is inaccurate. Consequently, we prefer to call this species Morafka´s desert tortoise. These common names will serve to exclude the species from other desert tortoises in the genus *Testudo*.

### Implications for conservation of westernGopherus

The most important implication of describing *Gopherus morafkai* is that Arizona and Mexico can no longer be considered to harbor a genetic reservoir for the Mojavian population of the desert tortoise, now exclusively defined as *Gopherus agassizii*. The recognition of *Gopherus morafkai* reduces the geographic range of *Gopherus agassizii* to about 30% of its former range (Van Devender 2002, Fig. 1.2); *Gopherus agassizii* now occupies an estimated 83,124 km2 of habitat (Fig. 2, also see model in U.S. Dept. of the Interior, Fish and Wildlife Service 2010a). *Gopherus agassizii*, which can now be referred to as Agassiz’s desert tortoise, has suffered tremendous population declines in the past 30 years (U.S. Dept. of the Interior, Fish and Wildlife Service 1994, 2010a). And much of the Mojave Desert does not offer habitat suitable for *Gopherus agassizii* (Hagerty et al. 2011). The taxonomic reduction of the species’ distribution can have dire consequences. Whereas species with broad distributions may survive population declines, those that have small distributions are far more likely to become extinct (MacArthur and Wilson 1963, 1967; Gilpin and Soulé 1986; Saccheri et al. 1998; O’Grady et al. 2006). Agassiz’s desert tortoise, currently listed as threatened under the Endangered Species Act of 1973 (as amended) (U.S. Dept. of the Interior, Fish and Wildlife Service 1990), may require a higher level of protection to ensure the level of management that would maximize its chances of survival.

A Recovery Plan was prepared for the Mojavian population (Agassiz’s desert tortoise) in 1994 (U.S. Dept. of the Interior, Fish and Wildlife Service 1994). Six recovery units were described in this Recovery Plan in an effort to capture ecological and genetic variation. The writers of the Recovery Plan also noted evidence of important ecological substructuring within the Western Mojave Recovery Unit, the largest and most heterogenous of the recovery units in terms of climate, vegetation and topography (U.S. Dept. of the Interior, Fish and Wildlife Service 1994). In an analysis of genetic differences within the Mojavian population, Murphy et al. (2007) confirmed that genetic substructuring existed within the Western Mojave Recovery Unit, with boundaries similar to those described in the 1994 Recovery Plan for western, central, and southern regions. The boundaries followed a major river, the Mojave River, as well as other climatic and ecological differences. Hagerty et al. (2011) confirmed the pattern reported by Murphy et al. (2007), although Hagerty and Tracy (2010) speculated that patterns reported by Murphy et al. (2007) were due to sampling bias. We think that the new genetic information from Murphy et al. (2007) and Hagerty et al. (2011) provide important support for updating recovery planning in the future.

### Conservation status of G. morafkai

Population declines for *Gopherus morafkai* within the USA appear to mirror those of *Gopherus agassizii* (Arizona Interagency Desert Tortoise Team 1996). In 2010, the U.S. Fish and Wildlife Service issued a determination that federal listing of the Sonoran population as threatened in the USA is warranted but precluded by other, higher priority species (U.S. Dept. of the Interior 2010b). The recognition of *Gopherus morafkai* is likely to hasten federal listing of the new species, *Gopherus morafkai*, in the USA. The Mojave population can no longer be considered to be a genetic reservoir for *Gopherus morafkai* or vice versa, and, unfortunately, the hybrid ex situ population involves a significant portion of tortoises presumed to be *Gopherus morafkai* (Edwards et al. 2010). These hybrids involve not only *Gopherus agassizii* but also *Gopherus berlandieri*. The genetic integrity of *Gopherus morafkai* may now be threatened by intentional release and escape of captive hybrids. As noted previously, natural hybrids occur in a limited portion of northwestern Arizona where Mojave and Sonoran ecosystems interdigitate (McLuckie et al. 1999). The hybrid zone appears to occur only in this area (Fig. 2). Little is known about the effects of hybridization on the native population, a topic that deserves attention. Adding to the concerns, *Gopherus morafkai* may contain two cryptic taxa in Mexico (Lamb et al. 1989). All species and populations in both Mexico and the USA would benefit from aggressive conservation action because of the potential for additional cryptic species in Mexico.

### Remaining problems

The questioned identity of *Gopherus lepidocephalus* has now been sufficiently answered to address its taxonomic status. The name is a junior synonym of *Gopherus agassizii*.Whether the holotype is a hybrid or not is taxonomically irrelevant because the maternal lineage had an origin in the Mojave Desert population. Nevertheless, three questions remain. First, is the holotype of *Gopherus lepidocephalus* a hybrid individual? This could explain its uniqueness (Ottley and Velázques Solis 1989) as well as it association with Sonora, Mexico (Crumly and Grismer 1994). Second, the question remains as to whether *Gopherus morafkai* consists of two forms that warrant recognition at the species level: Morafka’s desert tortoise and a potentially new Sinaloan thornscrub tortoise (Lamb et al. 1989). Currently, we are examining the spatial overlap of several genotypes at the eastern and southern boundaries of Sonoran desert scrub in Sonora, Mexico to better understand the evolutionary drivers responsible for shaping the genetic diversity of *Gopherus morafkai*, and to evaluate the possibility that the species is a composite of two cryptic species. Finally, it is critical to evaluate ontogenetic development in both species. This may vary geographically within species as well as with nutrition and other environmental parameters.

## Conclusion

Our investigation of the taxonomy of Agassiz’s land tortoise resolved many issues. The publication date has been given in error as 1863 since its first citation. The type series was likely collected by Cooper from near Soda Lake, California, and not elsewhere. Only one of the three original cotypes exists, USNM 7888, and it was designated as the lectotype. Our mtDNA sequence data from the lectotype confirmed that it was from California, not Arizona. Further, mtDNA sequence data from the holotype of *Gopherus lepidocephalus* placed its origin to the Mojavian population, rather than the Sonoran Desert of either Arizona or Mexico. Genetic, morphological and ecological data confirmed the existence of at least two species contained within *Gopherus agassizii*. The Sonoran population is named as a new species, *Gopherus morafkai*, Morafka`s desert tortoise. The recognition of *Gopherus morafkai* reduces the range of *Gopherus agassizii* to occupying about 30% of its former range. Given drastic population declines in *Gopherus agassizii* during the past few decades, it might be endangered.

## Supplementary Material

XML Treatment for Gopherus agassizii

XML Treatment for Gopherus morafkai
